# The experience of community first responders in co-producing rural health care: in the liminal gap between citizen and professional

**DOI:** 10.1186/1472-6963-14-460

**Published:** 2014-10-18

**Authors:** Anne Roberts, Amy Nimegeer, Jane Farmer, David J Heaney

**Affiliations:** The Centre for Rural Health, Centre for Health Science, University of Aberdeen, Inverness, IV2 3JH Aberdeen, UK; Institute of Health & Wellbeing, University of Glasgow, Glasgow, Scotland; Faculty of Health Sciences, La Trobe University, Melbourne, Australia

**Keywords:** Emergency medicine, Volunteering, Rural health, Community first response

## Abstract

**Background:**

The involvement of community first responders (CFRs) in medical emergencies in Scotland, and particularly in remote and rural areas, has expanded rapidly in recent years in response to geographical and organisational challenges of emergency medical service access. In 2013 there were over 120 active or developing schemes in a wide variety of settings. Community first responders are volunteers trained in First Person on the Scene (FPOS) first aid, administered prior to the arrival of an ambulance. Although there is limited literature which describes the role of first response, little academic literature has been published about the complexities of their specific role in both the community and organisational contexts.

**Methods:**

Here we reflect on data from two mixed-methods studies into the role of CFRs in Scotland.

**Results:**

We highlight findings that explore the liminal and complex role of the first responder as both ‘practitioner’ and community member, and how this contributes to a sense of communitas within the study areas. The rural context encompasses additional complexity in relation to the role of emergency care volunteer, having the highest levels of volunteering and this paper questions assumptions that rural areas, are more accepting of volunteerism.

**Conclusions:**

Complexities arising from the experience of blurred voluntary/practitioner boundaries emerge as a key feature of voluntary participation in medical emergencies in this setting.

## Background

Internationally, rural communities experience difficulties in accessing health care for reasons such as challenging geography, distance from service centres and reluctance of specialist health professionals to work in remote locations [1, 2]. Rapid access to emergency healthcare can be crucial for outcomes in critical health situations and rural residents are thus disadvantaged. Paradigmic change in public service provision from welfare state to self and community responsibility [3] have led to discussion, and increasingly realisation, of reconfigured rural health care delivery [4]. New policy messages encourage communities to build their capacity from within, to be resilient [5] and to “co-produce” basic needed services [6]. These messages are predicated on citizen’s rights to services being counter-balanced by their responsibilities to participate in production as well as consumption of services [7–9]. Exemplifying a new era of volunteering for co-production, Community First Responders (CFRs) act within the emergency care arena to provide local emergency care with ambulance services and other available health professionals.

Literature relating to community capacity suggests that rural areas may be ideal spaces for co-production as rural communities are richer in social capital [10] and have higher rates of volunteering [11], compared with urban communities. As the policy idea of co-production is relatively new, the literature tends to feature discussion of its potential rather than evaluation of its introduction.

We were interested to explore the experience of co-production in health care for rural lay persons. Timmons and Vernon-Evans [12] note there are few studies depicting the experiences of CFRs as literature tends to focus on their role in affecting health outcomes. This paper will examine the results of two research studies into first response in Scotland, in which a theme of liminality emerged as central to the role of first responders. Grounded in social anthropology of Van Gennep [13] and later applied to modern societies by Turner [14] the concept of liminality refers to transition, outside the everyday environment, for a period where the participant lacks social status or position within a particular community or group – an intermediate stage of being “in between”. In contemporary thinking, “liminality” may depict times of political or cultural change, and Turner argues liminality creates the idea of “communitas”, or an egalitarian sense of community and social togetherness that can occur within groups transitioning, in this case between lay community member and health professional.

In particular, this paper explores the experience of CFR liminality as lay persons exposed to helping fellow community members in stressful situations, with limited training and constrained scope of practice. We present our findings and discuss the emerging evidence, within this context, of the liminal first response role and argue that the foundation of such volunteering is in the creation of communitas.

This paper uses data from two mixed methods studies conducted in rural Scotland between March 2009 and September 2011. It presents the actual contribution of CFRs to community health and juxtaposes this with perceptions of CFRs and other community health stakeholders, about the CFR role. We use the data to depict CFR’s liminality and consider the implications for extending rural co-production.

CFRs fulfil an increasingly accepted community-based voluntary role in emergency service provision internationally, trained to provide basic level of ‘first person on the scene’ (FPOS) first aid training that includes patient resuscitation, basic wound care, and use of defibrillators. Evidence suggests that such trained community response during a medical emergency can affect patient survival, particularly for cardiac arrest [15, 16]. A systematic “chain of survival” during the first crucial minutes of an emergency response - early access, resuscitation, defibrillation and early advanced life support can increase an individual’s chance of survival [17]. Survival from defibrillation within three minutes of ventricular tachycardia/fibrillation (VT/VF) inception can be as good as 70-80%. In non-VT/VF arrest and VT/VF arrest without a defibrillator, survival using cardio-respiratory resuscitation (CPR) techniques alone can be as low as 2-8%, increasing to 20-30% in communities offering bystander CPR and rapid arrival of trained personnel [18], particularly those able to use Automated External Defibrillators (AEDs) effectively and in safety [19]. Therefore, CFRs can be an effective strategy for helping to reduce mortality in the community from cardiac arrest [20].

Diverse CFR models exist, with differences in types of emergency call attended, drugs CFRs are trained to administer, and additional clinical training. An English study showed diversity across different CFR schemes even within the same country; for example while some schemes responded solely to cardiac incidents, others attended diverse calls. Similarly, the study found that there was variation in the ability to provide medication other than basic oxygen between schemes [21]. Some schemes have contributed significantly to trauma care [22] where pre-hospital response times can be lengthy [23]. In Israel, for example, a dispersed model exists where volunteers carry a mobile telephone with an app that alerts the CFR nearest to the emergency [24]. Travelling predominately by motorbike for speedy response, this first responder model has had significant impact in Israel’s rural areas. In rural Australia there are volunteer systems where ambulance crew finance their own training and communities purchase ambulances and equipment when required [25]. This enables isolated communities to sustain feasible urgent care arrangements [26]. In spite of these benefits, introducing CFR schemes as a support to local health professionals, has sometimes occurred in situations of community protest. In an Australian community, O’Meara, Kendall & Kendall (2004) described that local people were placing unreasonably high expectations on small teams of resident health professionals and it took community development interventions for the community to accept a CFR scheme as part of local services [26]. The Healthcare Commission report [21] in England commented that CFRs benefit local communities in several indirect ways, including motivating people to be proactive in relation to their health, but there is little research evidence.

CFR schemes were first introduced to Scotland in 2002 and their number has increased since. Some schemes in Scotland have been established by the Scottish Ambulance Service (SAS), in areas identified as high need. Other CFR schemes have been established autonomously by members of the community. Scottish CFRs provide basic life-saving treatment as FPOS at selected emergency calls. When an appropriate call is received by an Emergency Medical Despatch Centre (EMDC), there is a synchronised response between the ambulance service and the CFR team, who then may arrive at the scene more quickly. Some schemes have elected to undertake further additional accredited training to FPOS intermediate level, which enables volunteers to attend a wider range of emergencies. There are now more than 120 schemes across Scotland [27]. At the time of these studies schemes were managed by five Community Resuscitation Development Officers (CRDOs), SAS employees whose role is to support and train volunteers. Some CFR schemes were supported financially by SAS, including for some, provision of a first response vehicle. Conversely, schemes established by communities were often reliant on local fundraising. Most schemes involved volunteers, although alternative models, such as retained ambulance services or joint first responder/fire service schemes were emerging. Volunteer numbers varied by scheme as did hours covered. Scottish CFR schemes existed in rural, remote and urban settings. Some flourished, while others experienced opposition, often because they were associated with perceived local service reduction [28].

The spread of CFRs has occurred in parallel with public service retrenchment and the emergence of policy encouraging greater societal input, and community participation and resilience; for example, increasing lay first responder schemes is cited as a pillar of Scottish rural health strategy [1].

While policy encourages citizen participation in basic service provision, research evidence delineates the ways in which challenges of co-productive involvement for volunteers are identified and addressed [29, 30]. First response to emergencies can be a traumatic role [31, 32]. Although the SAS strive to protect volunteers from extreme situations, in rural areas this maybe more difficult, with the added complication that people know each other, potentially bringing personal burdens of embarrassment, grief or responsibility. Policy depicts volunteering roles as positive, with benefits for communities and individuals. Such an approach adopts the principles of co-production – sharing information and encouraging collaborative decision making amongst service users and providers. Health research tends to focus on measuring objective health outcomes. The realities of the human experience within co-production, and what this means for increasing co-production, seem thus far to have been largely overlooked in the literature.

The few studies of rural people’s co-production experiences highlight the limitations of co-production, particularly given rural ageing populations. Studies in Canadian rural communities [31] have highlighted the almost unbearable burden of volunteering as services withdraw [33]. In Farmer & Bradley’s [34] study of impacts of developing rural social enterprises, they noted that the same people tended to take on more volunteering, rather than volunteering spreading more widely. This meant a heavy and stressful burden for a small number of residents.

Co-production therefore represents a paradigm change in service delivery. With volunteers on the threshold between lay citizen and skilled professional, such experiences require greater exploration in reality. Pragmatically, O’Meara et al. [35] highlight features of successful CFR schemes and Timmons and Vernon-Evans [12] explain volunteers’ motivations. We sought to assess if the features these researchers highlighted are more generalisable, and applicable to our Scottish context and highlight the new evidence which suggests CFRs occupy a liminal space in contemporary communities, between citizen and health practitioner.

## Methods

This paper uses findings from two studies. Study 1 (March 2009 – December 2010) evaluated the introduction of a CFR scheme in an isolated region with difficulties created by geography where the drive time to the nearest hospital with a major A & E department was more than 90 minutes. Study 2 (October 2010 – September 2011) investigated the contribution of six CFR schemes in urban, suburban and remote Scottish settings. Table 1 summarises the included schemes.

**Table 1:** **CFR schemes by urban rural classification** [36]

CFR scheme	Study number	No. of volunteers*	Reasons for inclusion in studies	Remote & Rural** (6 fold classification)
Scheme 1	2	8	Scheme described by the ambulance service as established.	Remote rural
Scheme 2	2	2	Remote island location with small number of volunteers.	Remote rural
Scheme 3	2	10	Co-response scheme with the Fire Brigade.	Rural area
Scheme 4	2	6	Newly established suburban scheme	Other urban area
Scheme 5	2	30	Larger number of volunteers, busy, urban scheme near to city area.	Other urban area
Scheme 6	2	21	Larger scheme, covering a collection of small towns	Small town
Scheme 7	1	9	Recently established scheme, remote & rural location.	Remote rural

*Number of volunteers “active” at the time of the study.

**The Scottish Government Urban Rural 6 folds Classification (Scotland).

“**Large Urban Areas**” Settlements over 125 000 people.

“**Other Urban Areas**” Settlements of 10,000 to 125 000 people.

“**Accessible Small Towns**” Settlements of between 3 000 and 10 000 people and within a 30 minute drive time of a Settlement of 10 000 or more.

“**Remote Small Towns**” Settlements of between 3 000 and 10 000 people and with a drive time of over 30 minutes to a Settlement of 10 000 or more.

“**Accessible Rural**” Areas with a population of less than 3 000 people and within a 30 minute drive time of a Settlement of 10 000 or more.

“**Remote Rural**” Areas with a population of less than 3 000 people and with a drive time of over 30 minutes to a Settlement of 10 000 or more.

Data collection during both studies were mixed methods. The aim was to capture the CFR activity data at the same time as gathering in depth, robust qualitative material. Included were stakeholder interviews (e.g. with representatives of national and local government, health authority, health professionals, and community members), and focus groups with individual CFRs. Routine anonymised data provided by SAS about callouts were analysed. Both studies were classified as service evaluations by local ethics committees (North of Scotland Research Ethics Service). All participants received information sheets clearly stating anonymity and confidentiality procedures and informed consent was provided by signed consent forms prior to participation.

### Routine SAS data

For Study 1, SAS activity data were obtained covering the first year of CFR scheme operation (2009–2010). For Study 2, SAS activity data for a six month period during 2011 for all six case sites were obtained. These were analysed to identify activity and case mix of contacts at all sites. CFR contacts are recorded on SAS contact sheets (including time of call, response, and arrival of ambulance, presenting complaint, and actions taken).

### Study interviews

Interviews for both studies were conducted either face-to-face or by telephone. Participants included purposely selected representatives from the Scottish Government (in the area of performance management for emergency medicine), Scottish Ambulance Service personnel, community engagement representatives from the Scottish Health Council, local after-hours service managers and General Practitioners (GPs). All individuals who were approached agreed to participate.

Interviews were conducted with community members, including formal community representatives (e.g. community council members) and ‘lay’ community members. Community members were initially contacted through community councils and pre-existing community groups (such as older people’s and parents groups). After initial contact had been made, snowball sampling [37, 38] was employed to contact further community members, often gaining introductions first from other community members to avoid unanticipated contact. One of the limitations of snowball sampling is that the sample it yields may not be representative [37, 39, 40] and it may be influenced by gatekeeper bias [41]. Hence, initial community contacts may intentionally exclude certain potential participants from their referrals. Conversely, Atkinson and Flint [40] note that snowball sampling provides a method for gaining access to closed or unknown communities and can be useful for ‘outsiders’ (such as ourselves as researchers) attempting to gain entrance. They also suggest the chain referral process may be more effective than other methods at fostering the inclusion of some who would be hard to identify by outside researchers.

Interviews were semi-structured, following a broad topic guide - but were informal enough to allow respondents to raise their own issues. Topic guides covered topics identified from existing CFR literature [42]: i) the benefits and limitations of CFR schemes, ii) obstacles and enablers, iii) views on the value of emergency response volunteering, iv) perceptions of potential further developments. In addition, lay community members were asked about their knowledge of the local CFR scheme, how it functioned, firsthand experience of using the scheme, and their thoughts about CFR schemes in local terms, and in general.

With consent, interviews in both studies were audio-recorded and transcribed verbatim. Analysis followed the framework approach [43] involving familiarisation with the data, identification of a thematic framework, indexing, charting, and finally mapping and interpreting the qualitative data. All transcripts were coded by AR and samples for verification, by AN, JF and DH. AR and AN conducted data analysis supported by NVivo 7 management software.

### CFR focus groups

CFR schemes were approached through their supporting SAS CRDO (Community Resuscitation Development Officer). Once initial agreement was obtained, researchers liaised with schemes to distribute information, obtain consent and arrange times and locations for focus groups. These took place at the usual meeting place for responder schemes (community hall, high school or GP Practice). Consent was obtained prior to arranging focus groups. With the explicit consent of all participants, groups were digitally sound recorded and transcribed verbatim. Discussions from both studies aimed to investigate CFR’s views about their role, training, support, relationships with other providers and community impacts. Focus group data were transcribed, coded and analysed as for the individual interviews, described above.

## Results

### CFRs contribution to emergency response

To establish the extent to which CFRs impact on community emergency services, we studied SAS data on CFR callouts. For Study 1, a remote and rural scheme, there were eight emergency calls for which a CFR was dispatched during the first year of the scheme’s operation. There was a total of 51 emergency call incidents in the area during this time (including daytime incidents attended by a GP, incidents inappropriate for CFRs, including road traffic accidents, and incidents stood down). Three of the eight were Category A (most serious). For Study 2, CFRs were dispatched for 200 calls during six months. Eighty-two (41%) were Category A. The three most common presenting complaints for CFRs were chest pain, possible acute heart problems, and unconscious/fainting. As seen from mean response times (Table 2), CFRs responded more quickly than ambulance crews. Evidence of their contribution to clinical care is unavailable, but, CFRs frequently provided the type of time-sensitive treatment that can have an impact on patient outcomes.

**Table 2:** **Ambulance and CFR response times**

	Mean response times (minutes)						
	Study 2						Study 1*
CFR Site	1	2	3	4	5	6	7
CFR attended after call-out	7.94	12.17	4.9	5.93	7.66	10.93	15.16
Ambulance/other resource attended after call-out	15.48	22.68	14.75	14.79	18.76	25.85	27.53
Minutes CFR arrived before ambulance	7.54	10.51	9.84	8.74	10.87	11.52	12.37
N (of total 206 calls)	17	33	2	74	50	24	6*

*The total number of calls in study 1 (n = 8), however only 6 were included to present in the data in the table above as 2 calls had no CFR “start time” documented on the data supplied.

For the total 206 calls, CFR response times were, on average, 10.19 minutes earlier than ambulance response times, 9.8 minutes in the urban schemes 4 & 5 and 10.14 minutes in the most remote rural schemes 1, 2 & 7. The current SAS response target is to reach 75% of Category A (life-threatening) emergency incidents within 8 minutes (excluding island responses). Therefore CFRs are contributing to the response gap in rural communities, providing basic FPOS life support that patients wouldn’t otherwise have.

### The CFR experience

Thirty-one interviews (22–75 minutes duration) were conducted. Fourteen interviews with local/national health authority/health providers and government representatives in emergency care and 17 with community members. Seven focus groups (one at each CFR scheme setting) were conducted. Key themes from this qualitative phase include understanding the CFR role; motivations for being a CFR; and issues around being of “the team” but not a professional. These are now explored.

### Being a CFR

Asked about their motivations, CFRs expressed enthusiasm for contributing to their community. They often stated their role as bridging the gap between health professionals and the community and providing support while awaiting ambulance arrival. Some had first aid knowledge:

“I became a first responder just to give something back to the community and also because I do basic life support training at work, to other people and I just thought it was a good way of maintaining it for myself and actually using it.” (Focus Group 2).

Experience of types of previous emergency situation influenced some:

“My dad took a heart attack and I had no idea what to do .....I want to try and help somebody because you’ve got no chance up here if you’re, say half an hour away from the hospital.....that’s the thing that pushed me into doing it”. (Focus Group 6).

“Well I joined because being a fire fighter you’re helping the community, so it’s just to, further help for the community and, you never know when you need the service yourself”. (Focus Group 5).

Most CFRs enjoyed the role and cited the opportunity to become emergency trained as an advantage. Supportive relationships amongst volunteers within their schemes and support from the wider ambulance service staff were reported. A small number wanted to use their CFR experience to help in getting paid healthcare-related employment.

CFRs discussed the drawbacks, including inconvenience to home life, especially if there were night-time callouts. Time away from family, stress and onus of ‘on call’ were all noted. In remote and rural areas, CFRs noted discomfort with potentially knowing the person you might have to assist. This issue was also discussed by community members in relation to being helped by a neighbour at a time of vulnerability. These challenges have been highlighted previously about rural healthcare professionals [44], however, they appeared to be complicated by community members’ perceptions of the CFR role.

### Confusion over CFR role

SAS employees and CFRs agreed on the scope of practice of CFRs’ emergency response duties, but community members were confused about the CFRs role. In some instances, community members thought a CFR attending was *instead* of an ambulance and professional ambulance staff, or regarded the introduction of CFRs with suspicion, as a precursor to professional service withdrawal. This was raised in remote and rural locations.

“Quite often the community see it as withdrawing, you know it’s a reason to withdraw the ambulance” (Interview 23, stakeholder).

There was evidence from rural and urban community members, that citizens did not understand the CFR role, were unaware of a local scheme and/or were unaware of the relationship between the CFR scheme and SAS, knowing what or who CFRs are, and not being aware that they were attended by a CFR:

“My husband and myself are both medical practitioners living in the area but have never heard of this scheme.” (Interview 4, community member).

“I don’t know of any first responder scheme in this village, if one exists there is a need for more publicity on who, what and when they can be used and the level of their skill and training.” (Interview 2, community member).

CFRs reported finding this confusion frustrating and, in one community in particular, it was found to divide the CFRs and community members who perceived the volunteers as colluding with service providers to remove local services. Conversely, community respondents also expressed feeling *“grateful”* and *“proud”* in relation to those who dedicate their spare time to benefit the community. They said their help was *“well-meaning, “brave”,* “*noble*” or *“community spirited”.* Such thoughts were most often expressed in remote and rural locations. A small number of participants described a personal experience of CFR attending and these participants valued CFRs.

“It was a much more rapid response than the ambulance crew could make and that was important for me. If I’d had to wait, I mean it was 45–50 minutes before the ambulance arrived, before anything was happening, so for me the rapid nature of that response was absolutely terrific, terrific.” (Interview 22, community member).

“I live in a very isolated part of the island so these people knew the name, knew the place and knew where they were going and that is very reassuring and I always knew that if another incident occurred that I would have that support and valued it and my elderly relative certainly valued it to the extent that she wanted to meet them in person when she was in a better place and personally thank them.” (Interview 18, community member).

During the focus groups CFRs expressed concern that community members lacked knowledge about the response process, particularly that CFRs only respond once an ambulance has been dispatched. CFRs perceived confusion in communities about reasons for introducing schemes. These views were most prominent in rural areas covered by CFR schemes 2 and 7. In Study 1 (scheme 7), CFRs were recruited whilst local health authority managers and community members were discussing ways of replacing an after-hours GP service. Community members were suspicious of CFRs, they thought their introduction meant there would not be a replacement professional after-hours service. CFRs in these rural schemes thought community members viewed volunteers as replacements for after-hours services.

“I’ve said to people, even if you do - at the end of the day - get a 24 hour doctor, we first responders will still be here…and they go “what?” so I say we are nothing to do with the doctor we are here for the ambulance service.” (Focus Group 2).

“I think that is the main issue, you hear the words ‘First Responders’, they thought the ambulance would be going…because they’ll think once the first responders are there that we’ll close the ambulance station.” (Focus Group 7).

All CFR volunteers in all schemes thought that more publically available information describing the CFR role and *“the point that the ambulance is on its way”* would help community members understand why CFRs volunteer and this may impact upon acceptance. Conversely, service providers and government representatives interviewed were generally knowledgeable about the specific contributions of CFRs.

CFRs also expressed varying confidence in fulfilling their role. Variation was in relation to the number of callouts experienced, (with rural CFRs called-out less than urban) and whether patients were personally known to the responder. CFR schemes required differing levels of commitment and availability of volunteers. Some schemes provide 24/7 cover, requiring a substantial commitment. CFRs do not administer medications, but volunteers that are also part of another agency (for example, the fire service) expressed desire to have the option of administering pain relief. Other CFRs expressed discomfort with the idea of additional clinical or drug administration responsibilities. As new schemes continue to develop and CFR roles evolve some first responders are advancing to more extended scope of practice. Ongoing developments and inconsistency between schemes appeared to add to confusion of CFR roles.

### Of the team, but not a professional

A commonly raised theme among CFRs and ambulance personnel was that while volunteers must act professionally according to a formal code of conduct and protecting patient information, they do not have the same emergency professional qualification that their colleagues have. One of the key issues regarding being *“in the team, but not a professional”* was confidentiality, and managing this both from an ambulance service perspective and within the community, as is illustrated by the following quotations:

“A big one is obviously about confidentiality, and that builds into the trust that the community places on them because when we first set up first responders schemes there was a concern from some that maybe the wrong type of person for the wrong reasons would be coming in and finding out about people at their most vulnerable so there’s a huge element of trust there and confidentiality is the big issue there.” (Interview 21, SAS manager).

“We don’t have any sanctions we can impose, they’re volunteers, so the only sanction we can impose actually is to say sorry we don’t want you to volunteer anymore.” (Interview 20, SAS manager).

“And I’ve heard other people say, just disparaging remarks for whatever reason about the idea of having people locally in the community coming into their homes, they might know, who they don’t really, they don’t see them the same as a paramedic or a doctor.” (Interview 16, community member).

Additional issues raised were CFRs not being allowed to administer medicines or to break the rules of the public road while attending a medical emergency (in the same way that an emergency vehicle could).

“I said but if we were responding to like a 999 situation, because we’re an emergency service, what’s the difference between our first responder car and the fire engine, because if you’re taking lights off the car, you’d be as well taking it off the fire engine.” (Focus Group 3).

Issues around CFRs being on the organisational periphery of services contributed to feelings of emergency care being provided on the “thin edge of the wedge” and being described as healthcare on the cheap:

“With the best will in the world, those girls are not trained to the same extent as technicians and paramedics that do it on a full time basis and it’s like community policing – it’s policing on the cheap – this is healthcare on the cheap and unfortunately it’s fraught with dangers….. I would prefer a proper and fully staffed NHS healthcare system.” (Interview 12, community member).

There was evidence that adhoc relationships had formed between some CFR schemes and local ambulance crew that resulted in equipment, emergency supplies, training/shadowing and post incident access to counselling, but these were informal and relied on local negotiation.

“In some of the areas as well, the relationships between, the voluntary responders and our crews are starting to develop so there’s, our crews will text them and say “Are you ok, you did well there, thanks for helping us out” and they’ll maybe make wee back and forward telephone calls, so there’s that type of support as well.” (Interview 26, Health Professional).

It was agreed that CFR schemes were not established or organised with consistent levels of support and training. Issues of governance remain challenging; one example involves the *“branding”* of CFRs, with the supply of uniforms and vehicles varying between schemes; some had ambulance service vehicles, while others used their own transport:

“Some schemes have cars…some schemes paid for them themselves so they’ll pay the lease, insurance and maintenance, tax costs. It’s marked as a Scottish Ambulance Service vehicle but it doesn’t have blue lights and so on so they don’t do an emergency response in it. They have a vehicle to take the kit to the patient. Some schemes have funded it themselves; some schemes use their own vehicles.” (Interview 20, SAS manager).

CFRs also commented that the lack of feedback about how patients fared was difficult to deal with. They were not formally informed about what happened to people after their first response assistance. This was challenging because they worked in the locality and may know the patient, their family or friends. Confidentiality prevented them from asking and yet they were often interested and concerned about fellow community members. Here, the boundaries between the health professional and the health volunteer are problematical. Sometimes CFRs got to know through word of mouth. Some described valuing this informal feedback:

“It’s very difficult, people are in pain or they’re not well, the contact we get is like one you had, the guy who had the heart attack, you meet him the next day, you may meet him in Tesco… I’ve met a couple of people out and said “how you doing?” and they look at you and they remember who you are, but that’s your feedback, we’ve got no real feedback.” (Focus Group 3).

“It would be nice to get some feedback, we have had feedback, but eh not them all, it depends how good the paramedic is that’s there and he gets in touch with out CRDO or his boss and says by the way that, these first responders did a good job you know.” (Focus Group 1).

Despite the challenges of dealing with such an ambiguous role at the threshold between professional and community member and for such a sensitive issue as emergency health care, the benefits of providing a link with the professional service and a community safety net were sometime expressed by those who valued community resilience:

“I think they can actually help build bridges between the, you know the front line you know emergency ambulance service managers, paramedics, technicians and the communities that its supporting.” (Interview 32, Health Professional).

## Discussion

Our two studies revealed that Scottish CFRs were able to respond to incidents more quickly than ambulance services. This is potentially beneficial to the impact on clinical outcome and thus, in health terms, CFRs were an important feature of local community health and security. CFRs were motivated by helping, perhaps having had personal experience of emergencies in the community, and wished to use their skills and, in some cases, to gain experience. This finding resonates with recent evidence about why CFRs volunteer [12]. The CFR role can be considered ‘liminal’ as these health volunteers lacked either a clear eminence as citizen in the community or status as a health practitioner during their voluntary experiences. In terms of lay contributions to the health system, CFR schemes exemplify a move to community members increasing role in co-producing basic services. Thus much can be learned from their experiences that can inform the increasing introduction of laypersons into what was formerly the domain of paid public service employees.

Co-production of services in rural areas, and CFR scheme in this instance, are underpinned by the concept of community resilience. It can also be argued that the foundation of such volunteering is contributing to a sense of communitas. Governments want communities to become less state-reliant and more self-reliant. In their enforced transition community members will need to move into different roles, flexibly and easily with a sense of communitas to achieve more self reliance and cause community members to unite and do things for themselves.

What this paper illustrates, however, is that the role of CFR also creates a liminal state for *individual* community members which causes them to pull together as a group within the community. While there is evidence that a sense of communitas is fostered *within* CFR groups, this paper also shows that the CFR role can foster a discomfiting liminality for participants – they are viewed as no longer wholly community members, but also not professionals, with the benefits that may bring. CFRs attract both gratitude and suspicion, and this very liminality brings stress for rural volunteers. The stress may be exacerbated in rural and remote areas if the introduction of CFRs is associated with withdrawal of statutory health services, due to the particularly high symbolic value that rural community members place on their traditional health services [45]. In other words, the presence of health professionals in rural areas is linked to perceptions of community sustainability. Therefore innovations which appear to threaten this sustainability will be met with suspicion.

This is not to say that CFRs were *not* found to contribute to rural communitas. There was evidence that community members reported feeling safer with CFR schemes in place, and that they were grateful for volunteers’ hard work. Likewise, CFRs cited a sense of community and reciprocity as motivations for volunteering. This paper does point out, however, that the CFR role is much more complex than portrayed in policy and SAS rhetoric.

CFRs navigate the tricky and stressful threshold between being a community member who knows their fellow citizens and a health worker who may have to assist in an emergency situation. It is this blurring of a traditionally professional role that often makes community members nervous. This could change as co-production roles become more embedded and thus is worthy of ongoing research.

As highlighted here, CFR schemes are not always welcomed. The introduction of rural CFRs occurs within a context of decreasing access to established service models, which if not always unequivocally effective, were embedded and normatively understood. Additionally, some ambulance personnel were suspicious of CFR schemes, perceiving them as threatening their jobs. This situation was compounded by poor communication and misinformation and inconsistent ‘branding’ that left community members and local health professionals confused as to where CFRs ‘fit’. The well-meaning community volunteer was placed at the centre of this paradigmic change.

CFRs are lay people who can actively save lives, but this liminality also brings the discomfort for some of feeling they could or should be doing more, and for others, of fear of being required to do more. Other discomfort arose from the potential of having to assist known people at times of extreme vulnerability and from being restricted in finding out about their health after an emergency incident. Thus, the remote and rural context may have exacerbated the sense of liminality for some volunteers, in the same way that it does for rural health care professionals [46, 47], in that it can be difficult to exist both as a ‘practitioner’ and as a community member when providing a service that includes confidentiality [48, 44]. It is also likely, however, that the liminality of CFRs was increased by poor communication and a lack of understanding of the CFR role. In areas with more mature schemes, there did appear to be less resistance to the concept as a whole.

Some challenges for our study participants may arise from distinct dissonance with the factors of a successful CFR scheme, as highlighted by O’Meara et al. [35]. In contrast to success factors highlighted in Australian research, several of the Scottish CFR schemes showed a lack of integration with mainstream ambulance services and variable and inconsistent resourcing.

Strengths of this study were its inclusion of CFR schemes across Scotland, and the use of mixed methods for data collection, thus allowing the voices of multiple types of stakeholders to be heard for verification of themes. The findings about inconsistent organisation and scheme type and discrepancies in communication, validate findings from an English study [21] about a rapidly growing service area.

### Limitations

A relatively small number of CFR schemes were included (n = 7) out of more than 100 schemes across Scotland. Each scheme was purposively selected to capture a variety of different geographical settings and stages of development. This selection process may have had an impact upon how fully comprehensive the results can be interpreted. Most of the CFRs in both studies had less than 3 years of experience in their role, primarily due to how newly established CFR schemes are in Scotland. CFR views and experiences of first response may change as schemes become further established.

Our studies were unable to link SAS activity data with clinical outcomes or measure the patient and carer perception of emergency care. The relationship between community resilience and uptake of co-production also requires further study.

## Conclusions

Community co-production is promoted as beneficial. While evidence presented supports that view, it is difficult for the individual to navigate and may place a high burden of stress, testing personal resilience by placing CFRs in zones of community and health practitioner contest, and requiring them to reconcile a complex role involving neighbour’s health with professional conduct standards, confidentiality issues and restrictions in scope of practice.

Evidence from other countries suggests that good leadership, information and support can assist CFRs. As the concept of CFR schemes embeds and schemes become more consistent in their organisation and presentation, perhaps CFRs’ liminal role will be more accepted and they will receive a greater sense of collective communitas from the communities and healthcare teams of which they form such a life-enhancing part.

## Abbreviations

CFR: Community first responder
FPOS: First person on scene
CRDO: Community resuscitation development officer
SAS: Scottish ambulance service
GP: General practitioner.

## References

[1] The Scottish Government (2008). Delivering for Remote and Rural Health: Final Report of the Remote and Rural Workstream.

[2] Commonwealth of Australia (2012). National Strategic Framework for Rural & Remote Health.

[3] Great Britain (2011). The Big Society, seventeenth report of session V1. House of Commons Public Administration Select Committee.

[4] Horne M, Khan H, Corrigan P (2013). People Powered Health: Health for People, by People and with People.

[5] Skerratt S (2013). Enhancing the analysis of rural community resilience: evidence from community land ownership. J Rural Study.

[6] Boyle D, Harris M (2009). Discussion Paper. The Challenge of Co-Production.

[7] Parks RB, Baker PC, Kiser L, Oakerson R, Ostrom E, Ostrom V, Percy SL, Vandivort MB, Whitaker GP, Wilson R (1981). Consumers as co-producers of public services: some economic and institutional considerations. Policy Stud J.

[8] Ostrom E (1996). Crossing the great divide: co-production, synergy and development. World Dev.

[9] Needham C (2007). The Reform of Public Service under New Labour: Narratives of Consumerism.

[10] Hofferth SL, Iceland J (1998). Social capital in rural and urban communities. Rural Sociol.

[11] Woolvin M (2012). Mapping the Third Sector in Rural Scotland: an Initial Review of the Literature.

[12] Timmons S, Vernon-Evans A (2013). Why do people volunteer for community first responder groups?. Emerg Med J.

[13] Van Gennep A (1960). (Translated by MB Vizedom and GL Caffee). Rites of passage. (Les rites de passage).

[14] Turner VW (1969). The Ritual Process: Structure and Anti-structure.

[15] Finn JC, Jacobs IG, Holman CD, Oxer HF (2001). Outcomes of out-of-hospital cardiac arrest patients in Perth, Western Australia, 1996–1999. Resuscitation.

[16] Smith LM, Davidson PM, Halcomb EJ, Andrew S (2007). Can lay responder defibrillation programmes improve survival to hospital discharge following an out-of-hospital cardiac arrest?. Aust Crit Care.

[17] Cummins RO (1993). Emergency medical services and sudden cardiac arrest: The chain of survival concept. Annu Rev Public Health.

[18] Nichol G, Myron L, Weisfeldt MD (2006). How to reduce sudden death in the community. Dialogues Cardiovasc Med.

[19] Hallstrom AP, Ornato JP (2004). Public-access defibrillation and survival after out-of-hospital cardiac arrest. N Engl J Med.

[20] Davies CS, Colquhoun MC, Boyle R, Chamberlain DA (2005). A national programme for on-site defibrillation by lay people in selected high risk areas: initial results. Heart.

[21] Healthcare Commission (2007). The Role and Management of Community First Responders. Findings from a National Survey of NHS Ambulance Services in England.

[22] **PRNewswire** [http://www.prnewswire.com/news-releases/united-hatzalah-of-israels-network-of-volunteer-emergency-first-responders-catches-worlds-attention-137891473.html]

[23] Murad MK, Husum H (2010). Trained lay responders reduce trauma mortality: a controlled study of rural trauma in Iraq. Prehosp Disaster Med.

[24] **Scattered aaviours: first aid that gets there first** *The Economist* 2012. [http://www.economist.com/node/21543488]

[25] O’Meara P, Burley M, Kelly H (2002). Rural urgent care models: what are they made of?. Aust J Rural Health.

[26] O’Meara P, Kendall D, Kendall L (2004). Working together for a sustainable urgent care system: a case study from south eastern Australia. Rural Remote Health.

[27] **Scottish Ambulance Service** [http://www.scottishambulance.com/YourCommunity/responders.aspx]

[28] Heaney D, Farmer J, Roberts A, Nimegeer A (2011). The Evaluation of the Introduction of a Community First Responder Scheme in Rannoch & Tummel.

[29] Kenny A, Hyett N, Sawtell J, Dickson-Swift V, Farmer J, O’Meara P (2013). Community participation in rural health: a scoping review. BMC Health Serv Res.

[30] Harrison-Paul R, Timmons S, van Schalkwyk WD (2006). Training lay-people to use automatic external defibrillators: are all of their needs being met?. Resuscitation.

[31] Haugen PT, Evces M, Weiss DS (2012). Treating posttraumatic stress disorder in first responders: a systematic review. Clin Psychol Rev.

[32] Mathiesen WT, Bjørshol CA, Søreide E (2014). Do bystanders need follow-up after performing CPR?. Resusc Vol.

[33] Skinner MW, Joseph AE (2011). Placing voluntarism within evolving spaces of care in ageing rural communities. Geo J.

[34] Farmer J, Bradley S (2012). Measuring the value of social organisations as service providers. Community Co-Production: Social Enterprise in Remote and Rural Areas.

[35] O’Meara P, Tourle V, Rae J (2012). Factors influencing the successful integration of ambulance volunteers and first responders into ambulance services. Health Social Care Community.

[36] **The Scottish Government Remote and Rural Classification** 1981.http://www.scotland.gov.uk/Topics/Statistics/About/Methodology/UrbanRuralClassification/Urban-Rural-Classification-2011-12

[37] Biernacki P, Waldorf D (1981). Snowball sampling: problems and techniques of chain referral sampling. Sociol Methods Res.

[38] Vogt WP (1999). Dictionary of Statistics and Methodology: A Nontechnical Guide for the Social Sciences.

[39] Kaplan CD, Korf D, Sterk C (1987). Temporal and social contexts of heroin-using populations: an illustration of the snowball sampling technique. J Ment Nervous Disord.

[40] Atkinson R, Flint J (2001). Accessing hidden and hard-to-reach populations: snowball research strategies. Social Res Update.

[41] Groger L, Mayberry PS, Straker JK (1999). What we didn’t learn because of who would not talk to us. Qual Health Res.

[42] Lewis J (2003). Design Issues. Qualitative Research Practice: A Guide for Social Science Students and Researchers.

[43] Yin R (2003). Case Study Research: Design and Methods.

[44] Iversen L, Farmer J, Hannaford P (2002). Workload pressures of primary health care professionals in rural areas. Scand J Prim Health Care.

[45] Prior M, Farmer J, Godden D, Taylor J (2010). More than health: the added value of health care to rural communities in Scotland and Australia. Health Place.

[46] Gillies J (1998). Remote and rural general practice. Br Med J.

[47] Lawrance R (2001). What symbolises rural and remote general practice: the practitioners’ perspective. Infront Outback.

[48] Bourke L, Sheridan C, Russell U, Jones G, DeWitt D, Liaw S-T (2004). Developing a conceptual understanding of rural health practice. Aust J Rural Health.

[CR49] The pre-publication history for this paper can be accessed here:http://www.biomedcentral.com/1472-6963/14/460/prepub

